# IL-1 Inhibitors in the Treatment of Monogenic Periodic Fever Syndromes: From the Past to the Future Perspectives

**DOI:** 10.3389/fimmu.2020.619257

**Published:** 2021-02-01

**Authors:** Hana Malcova, Zuzana Strizova, Tomas Milota, Ilja Striz, Anna Sediva, Dita Cebecauerova, Rudolf Horvath

**Affiliations:** ^1^Department of Paediatric and Adult Rheumatology, University Hospital Motol, Prague, Czechia; ^2^Department of Immunology, Second Faculty of Medicine Charles University and University Hospital Motol, Prague, Czechia; ^3^Department of Clinical Immunology and Allergology, Institute for Clinical and Experimental Medicine, Prague, Czechia

**Keywords:** IL-1, anakinra, canakinumab, rilonacept, CAPS, TRAPS, FMF

## Abstract

Autoinflammatory diseases (AIDs) represent a rare and heterogeneous group of disorders characterized by recurrent episodes of inflammation and a broad range of clinical manifestations. The most common symptoms involve recurrent fevers, musculoskeletal symptoms, and serositis; however, AIDs can also lead to life-threatening complications, such as macrophage activation syndrome (MAS) and systemic AA amyloidosis. Typical monogenic periodic fever syndromes include cryopyrin-associated periodic fever syndrome (CAPS), tumor necrosis factor receptor-associated periodic syndrome (TRAPS), mevalonate kinase deficiency/hyper IgD syndrome (MKD/HIDS), and familial Mediterranean fever (FMF). However, a number of other clinical entities, such as systemic juvenile idiopathic arthritis (sJIA), adult-onset Still’s disease (AOSD), Kawasaki disease (KD) and idiopathic recurrent pericarditis (IRP), display similar phenotypical and immunological features to AIDs. All these diseases are pathophysiologicaly characterized by dysregulation of the innate immune system and the central pathogenic role is attributed to the IL-1 cytokine family (IL-1α, IL-1β, IL-1Ra, IL-18, IL-36Ra, IL-36α, IL-37, IL-36β, IL-36g, IL-38, and IL-33). Therefore, reasonable therapeutic approaches aim to inhibit these cytokines and their pathways. To date, several anti-IL-1 therapies have evolved. Each drug differs in structure, mechanism of action, efficacy for the treatment of selected diseases, and side effects. Most of the available data regarding the efficacy and safety of IL-1 inhibitors are related to anakinra, canakinumab, and rilonacept. Other promising therapeutics, such as gevokizumab, tadekinig alfa, and tranilast are currently undergoing clinical trials. In this review, we provide sophisticated and up-to-date insight into the therapeutic uses of different IL-1 inhibitors in monogenic periodic fever syndromes.

## Introduction

Autoinflammatory diseases (AIDs) represent a heterogeneous group of rare disorders characterized by chronic and/or recurring systemic inflammation. AIDs are caused by dysregulation of the innate immune response and genetically are either monogenic or multifactorial in origin (1). In most cases, disease onset is in early childhood. However, late onset has also been observed. Due to the rarity of these heterogeneous disorders, patients may not be diagnosed at the time of symptom onset (2). Monogenic autoinflammatory diseases involve syndromes associated with periodic fevers, such as cryopyrin-associated periodic fever syndrome (CAPS), tumor necrosis factor receptor-associated periodic syndrome (TRAPS), mevalonate kinase deficiency/hyper IgD syndrome (MKD/HIDS), and familial Mediterranean fever (FMF). CAPS and TRAPS are typical autosomal dominant diseases, while MKD/HIDS, and FMF display hereditary transmission in an autosomal recessive manner. The clinical manifestations of AIDs involve recurring fevers and inflammation of the skin, synovia, pericardium, and pleura. Moreover, the gastrointestinal tract and central nervous system (CNS) may also be affected to variable degrees (1–3). The most severe clinical manifestation is secondary amyloidosis, which occurs in 2% to 25% of cases (4). Monogenic AIDs share pathophysiologic similarities with various multifactorial disorders, such as systemic juvenile idiopathic arthritis (sJIA) or Kawasaki disease (KD), which often results in similar clinical manifestations (5–8). All of these syndromes are predominantly characterized by an overproduction of IL-1 family cytokines; therefore, the use of IL-1 inhibitors represents a scientifically rational treatment approach. Currently, there are several anti-IL-1 therapeutic drugs (anakinra, canakinumab, rilonacept) that differ in structure, mechanism of action, effectiveness in treating selected diseases, and side effects. Other promising therapeutics are currently undergoing clinical trials (gevokizumab, tadekinig alfa, tranilast, dapansutrile) (9). In this review, we provide sophisticated and up-to-date insight into the therapeutic efficacy and safety of each IL-1 inhibitor in the treatment of monogenic autoinflmammatory periodic fever syndromes on the level of observational, open-label, randomized blinded controlled and registry-based studies.

## Cytokines of the IL-1 Family

**Figure 1:**
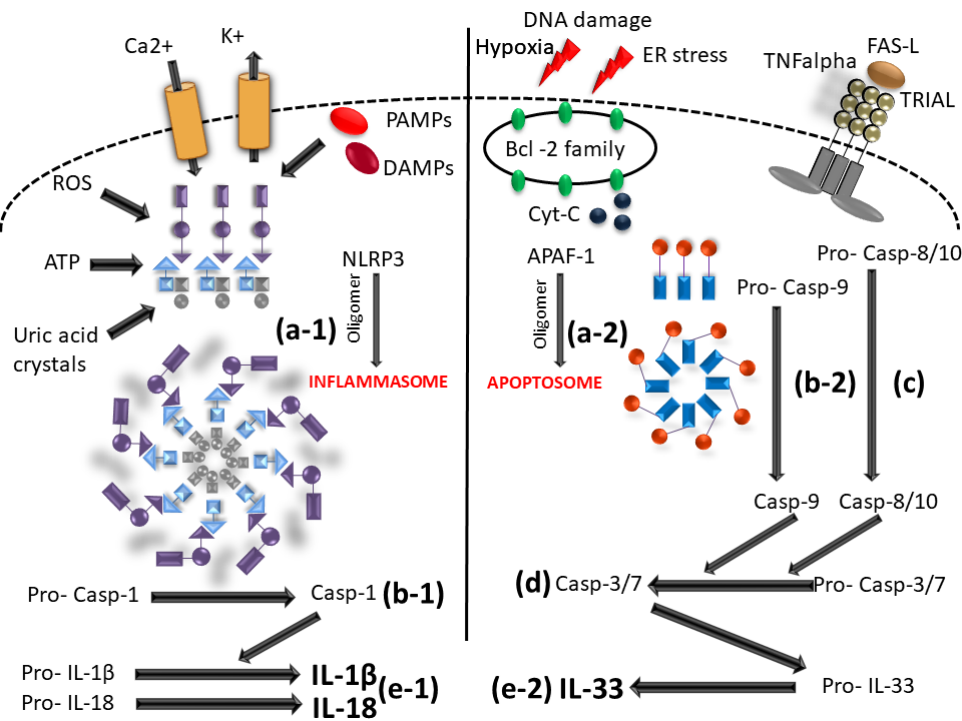
Scheme of caspase - 1/3/7 activation mediated by inflammasome and/or apoptosome. (a-1) initiation of NLRP3 oligomerization (by DAMPs; PAMPs; ROS; UA; potassium eflux; calcium influx); (a-2) APAF-1 oligomerization (upon intrinsic apoptotic way of activation by DNA damage; hypoxia; ER stress); (b-1) cleavage of pro-caspase 1 N-terminal region (inactive form) by the inlfammasome molecular complex; (b2) cleavage of pro-caspase-9 N-terminal region (inactive form) by the apoptosome molecular complex; **(C)** caspase-8/10 activaton by extrinsic apoptotic way of activation (by TNF; TRIAL; FAS molecules); **(D)** effector caspase-3/7 activation; (e-1) IL-1β and IL-18 activation from pro- IL1β and pro-IL18 inactive form by caspase-1; (e-2) IL33 mature form activation by caspase-3/7 (NLRP3, Nucleotide-binding oligomerization domain; leucine rich repeat and pyrin domain containing; DAMPs, Damage-associated molecular patterns; PAMPs, pathogen-associated molecular patterns; ROS, Reactive oxygen species; UA, Uric acid; APAF-1, Apoptotic protease activating factor-1; ER, Endoplasmic reticulum; TNF, Tumor necrosis factor; TRIAL, TNF-related apoptosis-inducing ligand).

**Figure 2:**
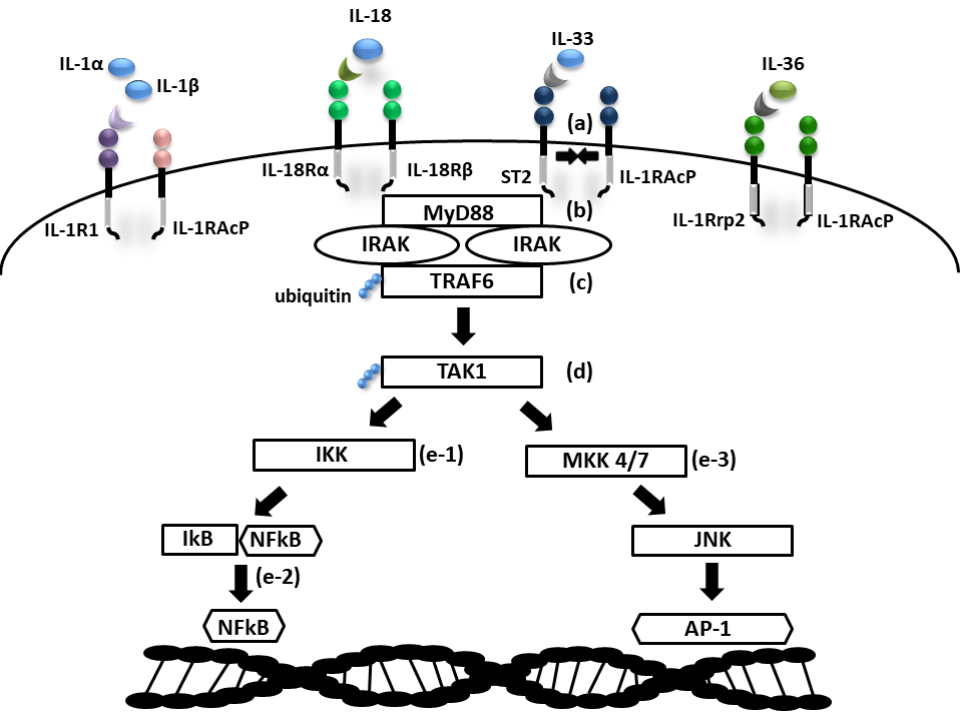
Scheme of IL1 receptor family signaling. (a) Cytokine binding on primary receptor initiates TIR domain containing accessory receptor recruitment necessary for signal transmission; (b) IRAK activation *via* MyD88 adaptor protein; (c) activation of TRAF6 ubiquitin ligase; (d) ubiquitin mediated activation of TAK1; (e-1) NFkB activation *via* IKK and (e-2) NFkB transition into nucleus; (e-3) AP1 activation *via* MAP kinases (MKK 4/7) and JNK (TIR- Toll/interleukin-1 receptor domain; MyD88, Myeloid differentiation primary response 88; IRAK, Interleukin-1 receptor associated kinase; (TRAF9, TNF Receptor Associated Factor 6; TAK1, Transforming growth factor beta-activated kinase 1; IKK, IkB kinase; IkB, NFkB inhibitor; NFkB, Nuclear factor kappa B; MAP, Mitogen activated protein kinase; JNK, c-Jun N-terminal kinase; AP1, Activator protein 1).

**Figure 3:**
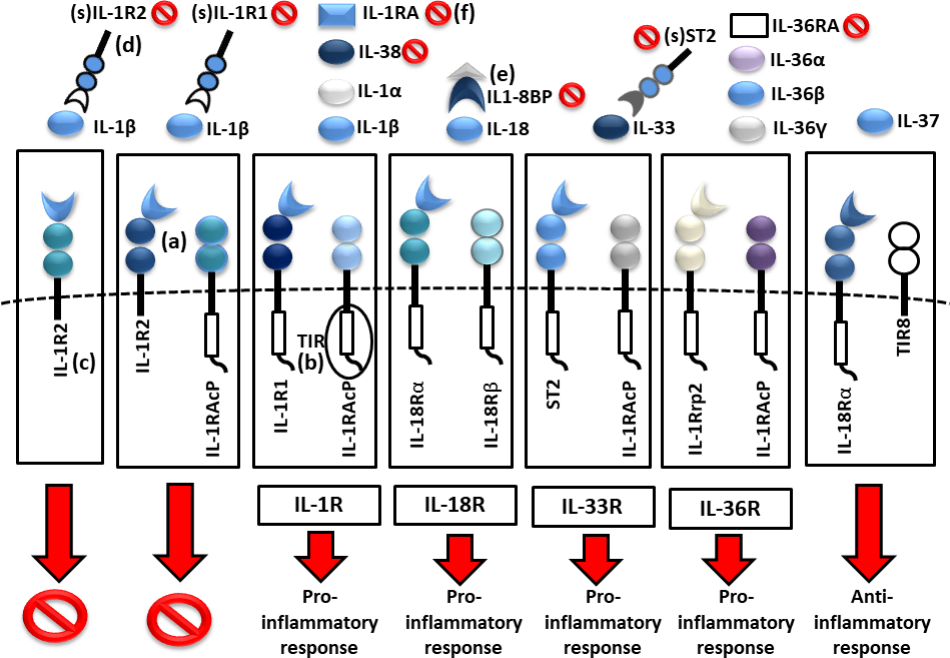
Scheme of IL1 receptor family structures and mechanisms of regulation. (a) cell membrane receptors structure of binary complexes—primary (IL-1R1, IL-18Rα, ST2, IL-1Rrp2) and accessory receptors (IL1-RAcP, IL-18Rβ), (b) signal transmission *via* TIR domains, (c) regulatory role of TIR-less receptors (IL1-R2) binding cytokines without signal transmission (inhibition 🚫), (d) regulatory role of soluble receptors (IL1-R1, IL1-R2, ST2) and (e) binding proteins (IL18-BP) binding cytokines without signal transmission, (f) inhibitory role of receptor antagonists (IL-1Ra, IL-36Ra, IL-38) (TIR, Toll/interleukin-1 receptor).

Cytokines in the IL-1 family are molecules that play a crucial role in the immune system functioning. Members of the IL-1 cytokine family bind to specific receptors and regulate the recruitment and activation of multiple immune cells. To date, there are 11 cytokines of the IL-1 family (IL-1α, IL-1β, IL-1Ra, IL-18, IL-36Ra, IL-36α, IL-37, IL-36β, IL-36g, IL-38, IL-33) divided into three subfamilies. These cytokines are closely linked to strong inflammatory response which is promoted by an activation of Caspases 1, 3, and 7. The activation of caspases leads to a formation of multiprotein oligomeric complexes and inflammasomes (caspase 1) or apoptosomes (caspase 3 and 7) (**Figure 1**). The signal from the IL-1 cytokine receptor is downstreamed after the cytokine binding through the Toll/interleukin-1 receptor (TIR) homology domain and the adaptor protein Myeloid differentiation primary response 88 (MyD88). MyD88 triggers a cascade of kinases that produce a pro-inflammatory signal leading to the activation of NFκB (10–12) (**Figure 2**). While most of the IL-1 cytokines participate in a strong inflammatory response, some bear anti-inflammatory characteristics. Cytokines, such as IL-37, IL-38, IL-36Ra and IL-1Ra, were shown to display different levels of anti-inflammatory properties (**Figure 3**). Nevertheless, the inflammatory properties of the IL-1 family dominate in the innate immune responses and the identification of strong IL-1 involvement in the pathogenesis of monogenic AIDs has revealed a great potential of IL-1 inhibitors in the treatment of these uncommon disorders (13)

## Inhibitors of the IL-1 Cytokine Family

There are currently three IL-1 inhibitors available for clinical use: anakinra, rilonacept, and canakinumab. Anakinra is a recombinant form of the IL-1 receptor antagonist (IL1-RA) that is physiologically expressed in humans. The mechanism of action includes the prevention of IL-1α and IL-1β binding to the IL-1 receptor. Anakinra, therefore, serves as a competitive antagonist of the IL‐1 cytokine and blocks its pro-inflammatory functions (14). Canakinumab is a human monoclonal antibody that specifically binds to IL-1β. The pharmacological effect depends on the blockade of the interaction between IL-1β and the IL-1 receptor. Thus, canakinumab prevents the activation of subsequent inflammatory responses (15). Rilonacept is a soluble receptor that predominantly neutralizes IL-1β but also neutralizes IL-1α. By acting as a soluble decoy receptor, rilonacept contributes to the reduction of inflammatory processes in diseases with predominant IL-1 cytokine pathology. While anakinra and canakinumab were approved for therapeutic administration in Europe, rilonacept is only available in the United States (16, 17). Among other promising therapeutics, gevokizumab, an IL-1β blocking monoclonal antibody, reduces the affinity of IL-1β to the IL-1RI/IL-1RAcP signaling complex leading to the modulation of cytokine imbalance in IL-1 mediated disorders (18). Tadekinig alfa is a human recombinant IL-18 binding protein (IL-18BP) that actively binds to free IL18 and thus prevents its binding to the receptor. Tadekinig alfa may provide clinical benefit in conditions that confer a high risk of a life-threatening complications, such as macrophage activation syndrome (MAS) (19). Tranilast, which was previously shown to inhibit IgE-mediated histamine liberation, binds directly to Nucleotide-binding oligomerization domain, leucine rich repeat and pyrin domain containing 3 (NLRP3). This specific binding blocks the formation of inflammasomes, which is crucial for caspase 1 activation and thus IL-1β production (20). Dapansutrile, is a novel orally active β-sulfonyl nitrile compound that serves as a direct selective inhibitor of NLRP3 inflammasome. Dapansutrile inhibits subsequent activation of IL-1β and is currently tested in the treatment of gout, autoimmune encephalitis and may also carry a large potential in the treatment of AIDs (21, 22). Other novel compounds, such as peptides and small-molecule inhibitors of the NLRP3 inflammasome, are tested in multiple pre-clinical studies as modulators of IL-1 mediated inflammation (23–25). Agents, such as diacerein, inzomelid or MCC950, are currently being tested in phase I/II clinical trials and as they affect also the IL-1 cytokine family, they might be evaluated also in the treatment of monogenic AIDs in the future (23).

Below we have addressed the main features of the monogenic periodic fever syndromes—CAPS, TRAPS, FMF, and MKD/HIDS, and extensively analyzed all available data on the anti-IL-1 treatment of these disorders in human clinical trials. We have separately introduced different aspects and results of observational studies, randomized placebo-controlled clinical trials and registry-based studies (**Tables 1A–D**).

**Table 1A:** Evidence for efficacy and safety of IL-1 inhibition in CAPS.

**CAPS**					
**Study population**	**Treatment**	**Study design**	**Follow up**	**Outcomes**	**Author**
18 NOMID pts. (age range 4-32 yrs.)	Anakinra (1-2 mg/kg/day)	Open label study	6 mo. (follow up)	• Immediate clinical and laboratory response in all pts •Improvent of hearing, resolution of headache, decrease in or disappearance of cochlear and leptomeningeal enhancement •Decrease in SAA, CRP and ESR •GC-sparring effect	Goldbach-Mansky et al. (26)
12 MWS pts. (5 children, 7 adults, age range 3-66.5 yrs.)	Anakinra 1–2 mg/kg/day (≥40kg: 100 mg/day)	Open label study	22–114 mo. (follow up)	•Rapid response to systemic and musculoskeletal symptoms in the majority of pts. •A decrease in CRP, SAA, and S100A12 levels •Proteinuria improved and hematuria resolved in all affected pts.	Kuemmerle-Deschner et al. (27)
43 CAPS pts. (36 children, 7 adults, age range 8 mo. - 46 yrs.)	Anakinra 0.5-1.5/mg/kg/day, adjusted to 1.5–2.5 mg/kg/day	Open-label	5 yrs. (follow up)	•Low AEs rate •Headache and arthralgia as the most frequent AEs •Headaches managed by increasing the anakinra dose	Kullenberg et al. (28)
7 pediatric CAPS pts. (5 MWS, 2 CINCA pts.)	Canakinumab 2 mg/kg (>40 kg:150 mg)	Open label study	126 to 463 days (follow up)	•Improvements in symptoms within first day •CRP and SAA levels normalized within seven days •CR in all pts. on canakinumab within seven days	Kuemmerle-Deschner et al. (29)
109 CAPS pts. (canakinumab-naive and 57 canakinumab roll-over pts.)	Canakinumab 2 mg/kg (>40 kg:150 mg)	Open label study	2 yrs.(follow up)	•CR in the majority if canakinumab-naive pts. •Higher canakinumab doses in younger patients and more severe CAPS as response predictors	Kuemmerle-Deschner et al. (30)
12 anakinra- and 14 canakinumab-treated MWS pts. (age range 3- 72 yrs.)	Anakinra 1-2 mg/kg/day (max 100mg); canakinumab 2 mg/kg (>40 kg:150 mg)	Open label study	23 to 115 mo. (median 52 mo. follow up)	•Reduced disease activity in both groups, higher response rate in canakinumab group •No superior effect in biologic-naive pts. •S100A12 as a marker of disease activity	Kuemmerle-Deschner et al. (31)
6 CINCA pts. (age range 11- 34 yrs.)	Canakinumab 2-4-8 mg/kg (>40 kg:150-300-600 mg)	Open label study	6 mo. - 24 mo. (extension phase)	•Persistent clinical and laboratory improvement in majority of pts. •Limited response to CNS manifestation •Favorable safety profile, only 1 SAE reported	Sibley et al. (32)
44 FCAS and 3 MWS pts.	Rilonacept 160 mg/wk.	Randomized, placebo‐controlled study with initial open-label phase	6 wks. open-label phase 9wks. randomized, placebo‐controlled phase	•Superior of rilonacept in reduction of disease flares •Improvement in clinical manifestation and laboratory findings •No SAE, ISRs and respiratory tract infection as the most frequent AEs	Hoffman et al. (33)
35 CAPS pts. with NLRP3 mutation (age range 4–75 yrs.)	Canakinumab 150 mg (≤40 kg: 2 mg/kg)/8 wks.	Randomized, double-blind placebo-controlled study with initial open-label phase and extension	8 wks. initial open-label phase 16 wks. randomized, double-blind placebo-controlled phase 24 wks. extension phase	•Superiority of canakinumab: CR or MDA in 89% of pts. upon a single dose, overall 96.8% response •SAA, CRP and IL-6 levels as a markers of disease activity • Impact of the therapy on quality of life	Koné-Paut et al. (34)
35 CAPS pts. (age range 4–75 yrs.)	Canakinumab 150 mg (≤40 kg: 2 mg/kg)/8 wks.	Randomized, double-blind, placebo-controlled study with initial open-label phase and extension	8 wks. initial open-label phase 24 wks. randomized, double-blind, placebo-controlled phase 24 wks. extension phase	•CR in 97% of pts. within 24 hours •Clinical and laboratory response during the entire double- blind period	Lachman et al. (35)
**Table 1B |** Evidence for efficacy and safety of IL-1 inhibition in TRAPS.					
**TRAPS**					
**Study population**	**Treatment**	**Study design**	**Follow up**	**Outcomes**	**Author**
4 children (age range 4–13 yrs.), 1 adult (age 33 years)	Anakinra 1.5 mg/kg/day	Open-label study	Range 4–20 mo. (mean 11.4 mo.)	•Prompt clinical and laboratory response to anakinra in all of the pts.	Gattorno et al. (36)
20 pts. (age range 7–78 yrs.)	Canakinumab 150 mg (≤40 kg: 2 mg/kg)/4 wks.	Open-label, proof-of-concept, phase II study	4 mo. administration, 5 mo. withdrawal, 24 mo. administration	•The median time of 4 days to CR •CR during the 4-month treatment period in almost all pts.	Gattorno et al. (37)
**Table 1C |** Evidence for efficacy and safety of IL-1 inhibition in FMF.					
**FMF**					
**Study population**	**Treatment**	**Study design**	**Follow up**	**Outcomes**	**Author**
7 pediatric pts. mean age 9.5 yrs.)	Canakinumab 150 - 300mg/4 wks.	Open-label	6 mo.	•Significant reduction in FMF attacks •Decrease in inflammatory markers (CRP, ESR, SAA) •No AEs reported	Brick et al. (38)
8 pts. (children and adolescent)	Anakinra 1-3 mg/kg/day, switch to canakinumab 2–3 mg/kg/day (max 150 mg)	Open-label	Range 3–28 mo. (mean 16.1 mo.)	•Efficacy of IL-1 inhibitors in colchicine-resistant pediatric patients •Switch to canakinumab due to non-compliance to anakinra •Unsuccessful withdrawal of colchicine in canakinumab treated patient •No SAE, 1 ISR reported	Basaran et al. (39)
20 pts. (median age 23yrs, range 14–50 yrs.)	Anakinra 100 mg/day, canakinumab 150 mg/8 wks.	Retrospective	Range 1–26 yrs. (median 12 yrs.)	•Significant reduction of flares on anakinra and canakinumab therapy •Effect on renal amyloidosis associated proteinuria •1 SAE, no allergic/skin SAE	Cetin et al. (40)
9 pts. (adolescents and adults)	Canakinumab 150 mg/4 wks.	Open-label	12 wks.	•Significant reduction (≥50% decrease) in number of flares in all pts. •High relapse rate upon treatment withdrawal	Gul et al. (41)
13 pts. (7 colchicine resistant, 6 FMF-related amyloidosis)	Anakinra 1 mg/kg/day (max 100 mg/day), canakinumab 2–4 mg/kg/6–8 wks.	Open-label	9–23 mo.	•A significant reduction in disease flares in both groups - colchicine resistant pts. and pts. with amyloidosis •Dose reduction required in patients with amyloidosis and chronic kidney disease •Limited effect of IL-1 inhibition on nephrotic syndrome	Özçakar et al. (42)
14 pts. (age range 13 -70 yrs.)	Canakinumab 150 mg/4-6-8 wks.	Observational	Retrospective follow-up 3-40 mo., prospective follow-up 10-30 mo.	•Complete CR in 79% pts. •Normalization of laboratory parameters in 91% pts., •8-week intervals as the optimal dosage regimen •Safe and well tolerated by all pts.	Laskari et al. (43)
15 pediatric pts.	Canakinumab 150 mg/8 wks.	Retrospective	Range 12–58 mo. (median 23.9 mo.)	•Complete CR in the majority of pts. by 12 mo. •No SAE or death	Gülez et al. (44)
14 pts. (mean age 24.4 yrs.)	Rilonacept 2.2 mg/kg/wk. (max 160 mg)	Randomized placebo-controlled clinical trial	4 mo.	•Reduced number of attacks, the duration of the attacks unaffected •No diferences between responders and non-responders at baseline characteristics •SAE related to FMF disease activity, ISRs as the most common AEs	Hashkes et al. (45)
40 pediatric pts.	Anakinra - 2mg/kg/day (max 100 mg/day), canakinumab 2mg/kg/dose (max 150 mg)	Registry-based study	3.87 yrs. (mean follow up)	•Decrease of CRP levels and reduced frequency of flares in anakinra/canakinumab group •Higher number of ISRs in anakinra group	Sag et al. (46)
25 pts. (mean age 6.8 yrs.)	Anakinra - 1mg/kg/day, canakinumab 2mg-4/kg/4 wks.	Retrospective	30 mo.	•Reduction of attacks and VAS •Successful switch from anakinra to canakinumab and vice versa •Improvement in quality of life, increased school attendance	Kurt et al. (47)
19 pediatric pts. (mean age 2.8 yrs.)	Canakinumab 3-6 mg/kg/4 wks.	Retrospective	up to 4.5 yrs.	•Reduction in number of attacks •Most of the patients previously treated with anakinra switched to canakinumab due to ISRs •No major AEs	Çakan et al. (48)
25 adult pts. (mean age 36.1–38.4 yrs.)	Anakinra 100mg/day	Randomized placebo-controlled clinical trial	20 mo.	•Reduced number of attacks •No SAEs reported	Ben-Zvi et al. (49)
61 pts. (mean age 18 yrs.)	Canakinumab 150–300 mg/4–8 wks.	Randomized placebo-controlled clinical trial with open-label phase	up to 72 wks.	•Reduction of flares and decrease in inflammatory markers (CRP, SAA) •No substantial changes in renal parameters •No SAEs	Ozen et al. (50)
**Table 1D |** Evidence for efficacy and safety of IL-1 inhibition in MKD/HIDS.					
**MKD/HIDS**					
**Study population**	**Treatment**	**Study design**	**Follow up**	**Outcomes**	**Author**
11 pts.(4 pediatric pts. age range 5–17 yrs.)	Anakinra 100 mg/day or 1 mg/kg/day/5–7 days	Prospective	4 mo.	•On-demand administration nas preferred way of application •Shorten of duration and reduction of severity of disease flares •No interference with vaccination observed	Bodar et al. (51)
9 pts. (6 children, 3 adults)	Canakinumab 300 mg (≤ 40 kg: 4 mg/kg)/6 wks.	Open-label	up to 24 mo.	•Rapid and persisting clinical and laboratory response •Disease flares after therapy withdrawal •Infections as the most common AEs •Up-reglated inflammasome/IL-1 and IFN signitures in active and untreated patients with down-regulation after therapy initiation	Arostegui et al. (52)
11 pts.	Anakinra 1–5 mg/kg, canakinumab 2–7 mg/kg	Retrospective	4–72 mo. (mean 15 mo.)	•Higher clinical and laboratory response rate in canakinumab group •Discontinuation of additional treatment •Higher prevalence of ISRs in anakinra group	Galeotti et al. (53)
63 FMF, 72 MKD, 46 TRAPS pts.	Canakinumab 150 mg (≤40 kg: 2 mg/kg)/4 wks.	Randomized placebo-controlled	40 wks.	•Efficacy of canakinumabin the FMF, MKD and TRAPS •Comparable response rate in 4-wk. and 8-wk. intervals, higher doses need in MKD •Infections as the most prevalent AEs, no death	De Benedetti et al. (54)
103 pts. (median age range 19 yrs., range 2–74 yrs.)	Anakinra 100 mg at the first signs of an attack	Registry- based study	Range 12–58 mo. (median 23.9 mo.)	• MTX, AZA, SAS, tacrolimus, dapsone, IVIG, montelukast, cimetidine, ranitided ineffective • Response to high dose of GC, anakinra and etanercept • Efficacy of etanercept in anakinra non-responders and vice versa	Van der Hilst et al. (55)
121 FMF, 94 CAPS, 113 TRAPS, 67 MKD pts.	Anakinra, canakinumab, rilonacept	Registry-based study	up to 22 mo.	•Lower response rate in EFR compared to the literature •IL-1 inhibitors as a treatment of choice in CAPS •IL-1 blockade as an therapeutic alternative in FMF, TRAP and MKD	Ter Haar et al. (56)

A list of observational; open-label; randomized placebo-controlled; and registry based clinical trials with IL-1 inhibitors in the treatment of 1A) CAPS; 1B) TRAPS, 1C) FMF; 1D) MKD (PFS, Periodic Fever syndromes; CAPS, Cryopyrin-Associated Periodic Syndromes; FCAS, Familial Cold Autoinflammatory Syndrome; MWS, Muckle-Wells Syndrome, TRAPS, Tumor necrosis–Associated Periodic Syndrome, FMF, Familial Mediterranean Fever, MKD, Mevalonate Kinase Deficiency; yr/s., year/s; mo., month/s; wk/s., week/s; pt/s, patient/s; s.c., subcutaneous; ESR, Erythrocyte Sedimentation Rate; CRP, C-Reactive Protein; SAA, Serum Amyloid A; S/AE, Severe/Adverse Event; ISR, Injection Site Reaction; MDA, Minimal Disease Activity; CR, Complete Remission; PGA, Patient Global Assessment; HRQoL, Health-Related Quality of Life; TNFa, Tumor Necrosis Factor alpha; GC, Glucocorticoids; MTX, Methotrexate; AZA, Azathioprim; SAS, Salazopyrin; IVIG, Intravenous Immunoglobulins; MA, Mevalonic Aciduria; EFR, Eurofever Registry).

## Methods

**Figure 4:**
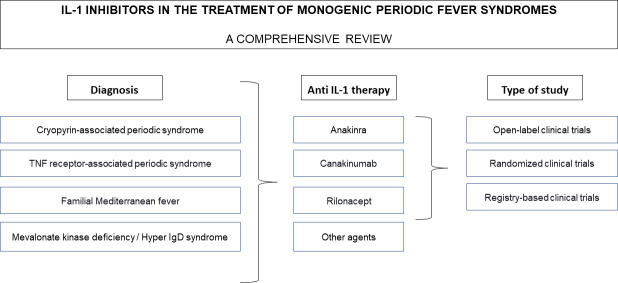
Flowchart diagram of the selection process. Anakinra, rilonacept and canakinumab in CAPS, TRAPS, FMF and MKD/HIDS were used as the key words in the search strategy, open-label and randomized clinical trials and registry-based studies were reviewed.

We conducted a comprehensive review of the literature on the efficacy and safety of IL-1 inhibition therapy including anakinra, rilonacept and canakinumab in CAPS, TRAPS, FMF and MKD/HIDS were used as the key words in the search strategy. Only English written and peer-reviewed reports published in indexed international journals until September 2020 were reviewed. Databases used for search included Medline/Pubmed, Scopus and Web of science. The selection process is summarized in **Figure 4**. The authors followed proposed guidelines for biomedical narrative review preparing (57).

## Cryopyrin-Associated Periodic Syndrome

CAPS include three disorders of clinically diverse severity: familial cold autoinflammatory syndrome (FCAS), Muckle-Wells syndrome (MWS), and the most severe chronic infantile neurological, cutaneous and articular syndrome/neonatal onset multisystem inflammatory disease (CINCA/NOMID). FCAS is typically associated with fever and symptoms, such as rash and intermittent episodes of myalgia, arthralgia, and conjunctivitis. These symptoms are usually triggered by the exposure to cold (58). MWS* *is a rare disease characterized by urticarial rash, arthralgia, and periodic episodes of fever and gradually progresses to sensorineural deafness and renal amyloidosis (59, 60). Of the CAPS syndromes, CINCA/NOMID is the most severe condition and is associated with a symptomatology triad: urticarial rash, arthropathy, and CNS disorders, such as chronic aseptic meningitis leading, possibly, to brain atrophy and sensorineural deafness (61). A typical facial appearance, such as a prominent forehead (frontal bossing) and saddle nose, is present in patients with CINCA/NOMID. Novel classification criteria of CAPS based on the genotype and/or typical clinical manifestation have been proposed by Eurofever/PRINTO (Paediatric Rheumatology International Trials Organization) (62) FCAS, MWS, and CINCA/NOMID are autosomal dominant diseases caused by a mutation of the NLRP3 gene, which causes the overproduction of IL-1β by cryopyrin inflammasomes (63). In clinical trials, CAPS therapy aims to block IL-1 specifically. A European initiative SHARE (Single Hub and Access point for pediatric Rheumatology in Europe) providing the treatment recommendations for the management of CAPS patients has highlighted the dramatically improved outcomes in CAPS patients on the IL-1 blockade (64). However, as the inflammation in CAPS patients is also driven by the mutations in NLRP3, novel molecules inhibiting NLRP3 inflammasome, such as tranilast and inzomelid, are currently also evaluated in phase I and II clinical trials (23, 25). Therapeutic potential of targeting NLRP3 priming and posttranslational modifications is yet to be uncovered (25).

To date, most of the evidence-based knowledge regarding the efficacy and safety of CAPS treatment is attributed to anakinra and canakinumab. Rilonacept, however, also shows promising results.

### Open-Label Trials

The initial report on IL-1 inhibition in the treatment of MWS was provided by Hawkins et al. (65). In this report, remarkable results were achieved in two MWS patients treated with 100mg/day s.c. anakinra which prompted further studies of IL-1 inhibition in CAPS (65). One of the first open-label studies with anakinra was performed by Goldbach-Mansky et al. (26), who analyzed its efficacy and safety in 18 CINCA patients. In all patients, a prompt clinical response (particularly disappearance of rash and conjunctivitis) was observed along with a decrease in inflammatory markers—erythrocyte sedimentation ratio (ESR), C-reactive protein (CRP) and serum amyloid A (SAA). The therapy had also a significant impact on the improvement of hearing (in 6 of 18 of patients), complete resolution of headache (8 of 18 patients), and decrease in or disappearance of cochlear (13 of 17 patients) and leptomeningeal (8 of 8 patients) enhancement. Additionally, intracranial pressures, protein levels, and white-cell counts also decreased significantly (12 of 12 patients). Anakinra had also GC-sparring effect. At month 3 after the initiation of the treatment, 11 patients underwent a short-term termination of the therapy for a maximum of seven days, which led to a disease flare in 10 patients within a median of 5 days. After resuming anakinra, all patients experienced a rapid response, which persisted at month 6 of follow-up period. Overall, most of the adverse events (AEs) included respiratory and urinary tract infections and injection site reactions (ISRs) (26). Prompt response to anakinra was also observed in an open-label study by Kuemmerle-Deschner et al. (27) focused on MWS patients. In this study, a treatment with anakinra led to a rapid and significant decrease in disease activity within 2 weeks (from DAS-MWS≥10 at baseline to 3.1 points, p = 0,0005). Clinical features together with laboratory parameters, such as erythrocyte sedimentation rate (ESR), were significantly reduced in 11 of 12 patients. A similar tendency was also observed in CRP, SAA, and S100A12 levels; however, these tendencies did not reach statistical significance. Overall, the therapy was well tolerated, and severe adverse events (SAEs) were not reported (27). The efficacy of anakinra in the treatment of CINCA and MWS patients was further reported in a number of case reports and case studies (66–73). Later, Kullenberg et al. (28) published the results of a prospective open-label representing the experience of anakinra in a large cohort of 43 CAPS. The study was primarily focused on safety profile of anakinra in two dosage regimens. An overall reporting rate of AEs was 7.7 events per patient year. These events included mostly headaches, arthralgia, and upper respiratory tract infections. No AE was reported with a frequency higher than 0.8 events per year SAEs occurred in 14 patients and were of infectious origin (28).

Based on the experience with anakinra, as a potent IL-1R antagonist, similar results were expected also with other IL-1 inhibitors—canakinumab and rilonacept. Kuemmerle-Deschner et al. (29) conducted a prospective open-label study of canakinumab in 7 pediatric patients with CAPS. Here, a complete response was seen in all patients within seven days after the first canakinumab dose (29). This study was followed by an open-label multicenter phase III study. This study cohort consisted of 109 CAPS patients with diverse phenotypes and clinical disease severities who had not been previously treated with canakinumab (canakinumab-naive) and 57 patients who had received previous canakinumab treatment (roll-over patients). Eighty-five canakinumab-naive patients developed a complete response which occurred within the first eight days in 79 of them. Remaining patients who did not achieve a complete response (defined as a global assessment of no or minimal disease activity and no or minimal rash, CRP and/or SAA levels within the normal range) displayed variable levels of symptomatic improvement. To achieve disease control, 40 patients required adjustment of the canakinumab dosage, and higher doses were required in pediatric patients than in adults. At least one AE occurred in 90.4% of the patients. In the pediatric group, six SAEs were reported (30). In addition, Kuemmerle-Deschner et al. (31) also performed a single-center open-label prospective observational study including pediatric and adult MWS patients, who were treated either with anakinra (5 children, 7 adults) or canakinumab (6 children, 8 adults). Both therapeutic approaches led to significant decreases in disease activity (from MWS-DAS 13 at baseline to 4 at the last follow up visit, p < 0.0001) and inflammatory markers. Looking at particular treatment options, remission was reached in 67% of anakinra, and in the canakinumab group, remission rates were even higher (93%). No superior efficacy was observed in IL-1-naive patients compared to patients with previous treatment. S100A12 levels reflected recurrence of disease activity and seemed to be a sensitive marker of subclinical disease activity (31). The effect of canakinumab was also evaluated by Sibley et al. (32) in 6 CINCA patients previously treated with anakinra during open-label phase I/II study. All patients were initially on anakinra at screening followed by its withdrawal for only 6–48 h due to early disease flares. Upon canakinumab initiation (150–300 mg, 2–4 mg/kg respectively) most of the patients improved clinically, as well as in the laboratory parameters, the response persisted for the entire study duration. However, none of the patients had CNS remission even with the maximal dose administered in the extension phase. Canakinumab was well tolerated, only 1 SAE was reported and was of infectious origin (32).

A pilot open-label study by Goldbach-Mansky et al. (74) with rilonacept demonstrated its efficacy and safety in 5 patients with FCAS. Only treatment-naive patients with active disease were enrolled. After the initial loading regimen (100mg in 3 consecutive days), a clinical benefit was observed within an hour. Maximal improvement in symptoms (significant improvement in daily symptoms from a score of 3.48 at baseline to 3.09, p < 0.05) and laboratory parameters (ESR, CRP, SAA) was noted at day 10 or earlier in all patients. However, the disease flares occurred between day 10 and 28 on the maintenance dose (100mg/week) and even further escalation of the dose (160–320 mg/week) did not lead to complete resolution, particularly mild joint pain and skin rashes persisted (74).

### Randomized Placebo-Controlled Clinical Trials

One of the first randomized, placebo-controlled, clinical trials with rilonacept in CAPS treatment was conducted by Hoffman et al. (33). Forty-seven patients with CAPS were enrolled and received an initial loading dose 320mg of rilonacept with a weekly maintenance dosing regimen 160 mg versus a placebo. At the end of the first part (week 6), 84% of the patients in the rilonacept group noticed a significant improvement in the clinical manifestations (compared to 13% in placebo group, p < 0.0001). The number of disease flares decreased from 8.6 at baseline to 0.1 (p = 0.0001) along with normalization of laboratory parameters (CRP and SAA). Additionally, 96% of the treated patients experienced at least a 30% reduction in disease activity. The efficacy was further supported by the results of the second part of the study, in which the response persisted in rilonacept treated group, while most of the patients in placebo group showed worsening of the disease symptoms. ISRs and respiratory tract infections were the most common AEs (33).

A randomized, double-blind placebo-controlled study by Koné-Paut et al. (34) assessed the effect of canakinumab in 35 CAPS patients, 89% of the patients reported absence of symptoms or minimal disease activity [evaluated Physician Global Assessment (PGA)] at week 8 of open-label phase. During double-blind placebo-controlled phase, this response persisted in those who maintained treatment with canakinumab. On the contrary, the treatment response was lost in the placebo group, only 25% of patients retained the response. However, responses were achieved in these patients shortly after resuming the therapy. The therapy had also significant impact on the patients’ quality of life. Levels of IL-6 reflected the disease activity. Severe adverse events were observed in only two patients (34). Similar results were obtained in another randomized study by Lachman et al. (35). A prompt response occurred in 34 of 35 (97%) patients after a single dose of canakinumab within 24 hours in a 8-week initial open-label phase. Afterwards, in a 48-week double-blind phase, all 15 patients continuing canakinumab treatment, maintained remission in comparison to 13 out of 16 patients (81%) in the placebo group, who experienced a disease flare in a median of 100 days after treatment withdrawal. Interestingly, no injection-site reactions were reported (35).

## TNF Receptor-Associated Periodic Syndrome

TRAPS is an autosomal dominant disease caused by a mutation in the TNFRSF1A gene encoding tumor necrosis factor receptor 1 (TNF-R1) (75). The median age of* *the disease onset* *is three years. Nevertheless, there have been reported cases of disease onset in the sixth decade of life. The condition is clinically characterized by long-lasting and recurrent episodes of fever and inflammation, which affect mostly the skin, gastrointestinal tract, serous membranes, joints, muscles, and eyes. Characteristic clinical manifestation and genetics have been also reflected in Eurofever/PRINTO (Paediatric Rheumatology International Trials Organization) classification criteria (62). The disease flares initially respond to corticosteroids; however, the response rapidly decreases over time, and uncontrolled flares further lead to the development of secondary amyloidosis (76). IL-1 has been described as playing a significant role in disease pathogenesis, and therefore, IL-1 blockade has become a treatment option for TRAPS (77, 78). Moreover, the SHARE initiative providing evidence-based recommendations for the treatment of TRAPS demonstrated that the IL-1 blockade is beneficial in the majority of cases (64).

### Open-Label Trials

TRAPS patients have been already successfully treated with anakinra in 2008. An open-label study by Gattorno et al. (36) evaluated the efficacy and safety of anakinra in 5 patients with TRAPS. All patients responded promptly and remained symptom-free. The levels of inflammatory markers, including SAA, were reduced in all patients as well. According to the study protocol, the therapy was given to all patients for 15 days, and then the treatment was withdrawn. All patients experienced a disease flare after a few days (ranging from 3–8 days). After treatment resuming a disease control was promptly achieved again. Afterwards, the patients were observed for 11 months, and no fever episodes or other symptoms associated with TRAPS were reported. The only adverse events were skin symptoms, such as rash, pain, and pruritus, which occurred within the first week of treatment (36).

In another open-label phase II study by Gattorno et al. (37) canakinumab was testing in 20 TRAPS patients with active disease (active recurrent or chronic TRAPS) as a potential treatment modality. Again, the response to canakinumab was fast and led to clinical remission (defined as PGA ≤1 with CRp < 10 mg/liter and/or SAA <10 mg/liter) within 3 to 8 day in all patients with evident impact on patients´ quality of life. Furthermore, either complete or partial laboratory remission was observed in 95% of the patients. Scheduled canakinumab withdrawal at month 4 resulted in disease relapse with the median time 91.5 days (ranging from 65 to 117 days). Generally, the therapy with canakinumab was well tolerated. Despite the fact each patient reported at least one AEs, SAEs occurred in 7 patients only (37).

### Randomized Placebo-Controlled Clinical Trials

A single randomized double-blind, placebo controlled clinical trial aimed to evaluate the efficacy of canakinumab in TRAPS patients. This study by De Benedetti et al. (55) with subsequent randomized withdrawal/dosing frequency reduction and open-label long-term treatment epochs has been completed in July 2017 (see details below, section 7.2.). Currently, there are no ongoing randomized clinical trials with IL-1 inhibitors in TRAPS patients.

## Familial Mediterranean Fever

FMF is a hereditary autoinflammatory disease with the highest prevalence among monogenic AIDs. This mostly autosomal recessive disease is characterized by recurrent episodes of fever and polyserositis. The episodes of fever are often accompanied by abdominal pain due to peritoneal inflammation and by musculoskeletal involvement including arthritis and febrile myalgia. Arthritis is usually present in around 70% of the patients in a form of transient non-erosive monoarthritis affecting large joints. The progression to chronicity is observed only in a limited number of patients (38, 79) The intervals between fever episodes vary among each individual and may endure from weeks to years. Long-term complications are also frequently prevalent. The most severe complications include amyloidosis which in some cases may lead to the kidney failure (80, 81). The detailed pathogenesis of FMF remains largely unknown; however, the MEFV gene has been previously associated with the disease development. The MEFV gene encodes pyrin, and a mutation of MEFV results in an uncontrolled IL-1 production (80). Due to the clinical complexity of the FMF disease, Eurofever/PRINTO (Paediatric Rheumatology International Trials Organization) group and the European League Against Rheumatism (EULAR) have presented novel classification criteria and evidence-based recommendations to guide rheumatologists in the treatment of FMF patients (62, 82)

Two main goals have been proposed. The first goal aims at preventing the clinical attacks. The second goal is to suppress chronic inflammation and elevation of the SAA protein. According to EULAR recommendations, IL-1 blockade should be considered especially in patients that fail to respond to colchicine. Despite the high effectivity of colchicine, the unresponsiveness has been described in around 5–10% of FMF patients. However, the definition of “colchicine resistence” remains elusive and different criteria have been use across the studies. The mechanisms underlying the colchicine resistance are not known. Another aspect limiting the use of colchicine is also intolerance reported in 2–5% of patients, mainly due to gastrointestinal adverse events (83, 84). The inadequate responses to colchicine have been described throughout various autoinflammatory disorders (85, 86).

Therefore, the treatment of colchicine resistant FMF patients remains a challenge and IL-1 blockade represents a promising treatment option.

### Open-Label Trials and Observational Studies

An open-label single-arm pilot study was presented by Brick et al. (39). In this study, 7 children with colchicine resistant FMF were treated with s.c. canakinumab 2mg/kg for a total of six months. A significant reduction (50% and more) in the FMF attacks was observed in almost all patients. The therapy also led to significant changes in laboratory parameters (CRP, ESR, SAA). Three patients did not report a single disease attack during the study period. In two patients, the canakinumab dose had to be escalated due to low treatment response. The proportion of complete responders, however was high, and no SAEs were observed (39).

Four different studies evaluating anti-IL-1 inhibitors were presented in 2015. An open-label study conducted by Basaran et al. (41) included eight colchicine- resistant children and adolescent patients with FMF who were treated with anakinra. Despite the fact that clinical remission with no attack was achieved in seven of eight patients, in three patients a switch to canakinumab was performed due to a non-compliance at month 6. After 3 months of the therapy, the unsuccessful attempt with colchicine cessation led to re-appearance of low-grade fever, abdominal/chest pain and elevation of inflammatory markers and colchicine therapy had to be reinitiated. Generally, anti-IL1 therapy was well tolerated. There were no SAE reported, and only one patient developed a ISR (41). Additionally, Gul et al. showed, that short-term therapy with canakinumab withdrawal (at week 12) caused disease relapse in five of nine patients within 31–78 days (40). Apart from open-label studies, the findings endorsing anakinra and canakinumab therapy were also confirmed in multiple retrospective studies. A retrospective study by Cetin et al. (42) aimed to evaluate the use of anakinra in 12 and canakinumab in 8 FMF patients upon colchicine failure. Both therapeutic options led to a significant reduction of attacks—from mean 1.5 (range 1–4) attack/month at baseline to 0 (range 0–3)/month at the end of follow up and from 18 (range 2–50) attacks/year to 1 (range 0–24)/year (p = 0.001). Surprisingly, the authors also reported the effect of the therapy on the level of proteinuria in two, pediatric and adult, patients with biopsy-proven renal amyloidosis, which usually respond in very limited manner. Furthermore, there were no allergic or skin SAEs. Only one severe infection was reported in the anakinra treated patient (42).

Another study was presented by Özçakar et al. (44). Thirteen subjects out of the cohort of 330 FMF patients were treated with IL-1 inhibitors, anakinra and canakinumab, due to colchicine resistance or amyloidosis. Treatment led to a significant reduction in disease flares from a median of 36 attacks/year (range 12–16, p = 0.018) at baseline to 3/year (range 0–4, p = 0.018) in addition to decreases in acute phase reactants, including the ESR (from pretreatment median 69 mm/h to posttreatment 23 mm/h, p = 0.018) and CRP level (from median 90 mg/liter to 2.7 mg/liter, p = 0.018). There was similar response in both group however, IL-1 inhibition had only a limited effect on nephrotic syndrome. All patients with amyloidosis and chronic kidney disease required dose reduction to 1 mg/kg/day and maximal dose of 100 mg/day three-times a week (44). Comparable outcomes of canakinumab and anakinra efficacy and safety profile were also reported in other retrospectively analyzed cohorts of FMF including recently published study by Gülez et al (43),Laskari et al. (87), and Kucuksahin et al. (88). Moreover, Laskari et al. also analyzed different dosage intervals of canakinumab–4, 6 and 8 weeks. Based on the results, the authors suggested to initiate the therapy in 8-week intervals. Shorter intervals should be considered in cases of persisting disease activity. Kucuksahin et al. then demonstrated limited responses to anti-TNFα therapy in FMF patients. On the other hand, in the treatment of articular involvement specifically, more profound responses were seen in FMF patients undergoing anti-TNFα treatment than IL-1 blockade. These results were supported by a number of case series by Eroglu et al. (47)

A study by Kurt et al. (48) has evaluated the clinical and laboratory parameters, as well as the quality of life, in 25 colchicine-resistant FMF children treated either with canakinumab or with anakinra. Out of eleven patients treated with anakinra, six successfully switched to canakinumab due to noncompliance and side effects. As opposed, two patients receiving canakinumab were switched to anakinra because of increased frequency of attacks. In both study groups, the anti-IL-1 blockade treatment has been shown to significantly improve the quality of life in colchicine-resistant disease and to increase school attendance in FMF children. A significant reduction in the frequency of attacks and improvements in the VAS levels were observed in both anti-IL-1 treatment groups (48).

Çakan et al. (89) further reported the efficacy of canakinumab therapy in 19 colchicine-resistant pediatric FMF patients. In these patients, the mean number of attacks per year with colchicine treatment it was 8.2 and dropped to 0.1 on the anti-IL-1 therapy. In 14 of 19 FMF patients, anakinra was used before canakinumab. The main reason for the IL-1 therapy switch was reported to be the painful daily injections (89).

Multiple other observational studies, case series and conference abstracts have reffered to the efficacy of IL-1 inhibition in FMF patients. Despite so far promising results, Kuemmerle-Deschner et al. highlighted the need for properly designed prospective or controlled studies to conclude the superiority of one anti-IL-1 therapy over another (45).

### Randomized Placebo-Controlled Clinical Trials

A first randomized placebo-controlled study evaluating the effectiveness and tolerability of anti-IL-1 agent rilonacept in FMF patients was performed by Hashkes et al. (49). In this study 14 FMF patients resistant to or intolerant of colchicine were divided into 4 groups with different rilonacept–placebo sequences. In total, more than a half of patients responded to treatment and the number of attacks significantly decreased from 3.3/month to 0.77/month (p = 0.027). However, the proportion of time in attack in rilonacept and placebo group remained unaffected. There were also no differences between responders and non-responders in the baseline characteristics. Together, 9 SAEs were reported, 7 of them were related to disease activity in FMF and occurred in non-responders, remaining two SAEs included respiratory tract infections. Additionally, the majority of AEs were ISRs (49). Another randomized placebo-controlled study by Ben-Zvi et al. (50) focused on the evaluation of anakinra efficacy in colchicine-resistant FMF. In this study, 12 patients that were randomly assigned to the anakinra group experienced significantly lower number of attacks in all assessed sites (1.7 versus 3.5 attacks per patient per month). Five out of 13 placebo patients discontinued the study due to the treatment failure, however, no SAEs were reported in either of the groups. Anakinra was well tolerated and provided a superior efficacy over placebo (50).

A CLUSTER study, a 16-week randomised phase III clinical trial with 72-week open-label period presented by Ozen et al. (46) also measured the long-term treatment responses to canakinumab in colchicine-resistant FMF patients. In this 72-week long study, 61 patients were enrolled. The canakinumab treatment significantly affected the disease control which was reflected mainly in the reduction of disease flares and the decrease of the CRP levels. 58.3% of the patients exprerienced no flares and 38.3% of the patients experienced one flare. The median SAA levels remained over the limit of normal (10 mg/liter) but under the 30 mg/liter threshold. Substantial changes in median creatinine clearance values were not observed during the study and thus, it has not been elucidated whether canakinumab treatment can also prevent amyloidosis and renal complications. No SAEs were reported during the study period and in conclusion, the canakinumab treatment was shown to bear a potential of controlling disease in FMF patients as a long-term therapy (46).

### Registry-Based Studies

The most recent data regarding anti-IL-1 treatment were reported by Sag et al. (90). The study focused on 40 colchicine-resistant pediatric FMF patients from HELIOS registry that were treated with anakinra and canakinumab. Anakinra was used as the first-line anti-IL-1 treatment in all patients (34 continuous and 6 on-demand use). Nevertheless, 22 patients were switched in a median 9.5 months to canakinumab treatment. Patient responses were evaluated at month 6 and at the last visit. Overall, 6 patients were treated with anakinra and 28 with canakinumab at the end of the follow up. Anti-IL-1 therapy (anakinra/canakinumab) resulted in a significant decrease of the CRP levels and led to a reduced frequency of flares. The most frequent AEs were ISRs, which occurred in 11 patients on the anakinra treatment which is in correspondence to higher incidence of ISR observed also in the other trials with anakinra treated patients regardless the indication. All these patients had to be switched to canakinumab. Other reported AEs included infections, leukopenia and thrombocytopenia (90).

## Mevalonate Kinase Deficiency/Hyper IgD Syndrome

MKD/HIDS in an autosomal recessive disorder caused by a genetic mutation resulting in an autoinflammatory condition. Mutations are found* *in the MVK* *gene* *encoding mevalonate kinase, which is directly associated with cholesterol and isoprenoid biosynthesis. The disease onset is usually within the first postnatal year and is characterized by recurrent fevers (every 4–6 weeks), generally lasting from 3 to 7 days (51, 91). Clinical symptoms range from milder MKD/HIDS to its most severe manifestation called “mevalonic aciduria,” which is associated with intellectual disability. The febrile episodes are accompanied by symptoms such as abdominal pain, diarrhea, vomiting, arthralgia, lymphadenopathy, heterogeneous skin lesions, and oral ulcers. Similarly to other major monogenic periodic fever syndromes, Eurofever/PRINTO (Paediatric Rheumatology International Trials Organization) recently published classification criteria including typical symptoms and genotype (62). The attacks are often triggered by vaccinations, infections, emotional stress, trauma, or surgery. The pathophysiological background involves increased mevalonic acid and further activation of small GTPases, resulting in IL-1 overexpression. Therefore, the short-term blockade of IL-1 may be useful for preventing inflammatory attacks (1). Also, the SHARE initiative has supported the short-term IL-1 blockade in terminating the inflammatory attacks and recommended the anti-IL-1 therapy to prevent the side effects of corticosteroids (64). To date, several clinical trials have been performed on the treatment of MKD/HIDS with Il-1 inhibitors, however, further studies are needed.

### Open-Label Trials and Observational Studies

In a prospective observational study by Bodar et al. (52), the effectivity and safety of on-demand application of anakinra was evaluated in 7 patients, who did not prefer continuous treatment. In all patients anakinra dramatically shortened the duration and reduced the severity of the attacks. The improvement was achieved in cases where anakinra was administred within 24 hours since the onset of the first symptoms. Nevertheless, on-demand treatment did not decrease the number of flares. Despite of the reduced efficacy in frequent users, the response was restored upon 2-month withdrawal and no patient developed persistent loss of efficacy. SAEs were not reported. Again, ISRs were the most common AEs. Interestingly, the authors also evaluated the impact of anakinra on post-vaccination response, which was adequate in all vaccinated patients. Therefore, anakinra provides safe and effective prevention of flares in those patients requiring vaccination. Based on these results, the authors advocate on-demand application of anakinra in patients with lower number of attacks or without long-term complication such as amyloidosis (52).

Arostegui et al. (53) investigated the efficacy, safety and gene expression profile of 10 MKD patients with no or poor disease control at baseline in an open-label phase II study with canakinumab. In a 6-month phase, all patients experienced a rapid response after first application within the median time 3 days (range 1–5) to resolution. In following withdrawal period (P1), 7 patients experienced an attack within median time 110 days (range 62–196) after the last administration of canakinumab. The median time of the attack duration was 3–4 days (range 2–10). Clinical response was also accompanied by a normalization of the CRP level. The efficacy persisted also in the 24-month extension period. The most common AEs were of infectious origin. The therapy was also associated with a decrease in leukocyte and neutrophil counts. Moreover, the analysis of gene expression profile showed up-regulated inflammasome/IL-1-related and IFN-inducible signatures in untreated and active patients. Interestingly, the dysregulation of both signatures has not been observed in the other autoinflammatory diseases. The initiation of canakinumab therapy led to their down-regulation (53).

The efficacy and safety of anakinra and canakinumab were compared in an observational study by Galeotti et al. (92) in a cohort of 11 MKD patients. Complete clinical response (defined as absence of attacks and inflammatory syndrome) was achieved only in a single patient out of nine on anakinra treatment compared to three of six in the canakinumab group. Additionally, four patients treated with anakinra had to be switched to canakinumab. Favorable response to canakinumab was also seen in normalization of laboratory parameters. Both therapeutic modalities allowed a discontinuation of the additional medication (92). The very similar results were also obtained in the Japanese nationwide survey conducted by Tanaka et al. (54).

### Randomized Placebo-Controlled Clinical Trials

The randomized placebo-controlled clinical study by De Benedetti et al. (55) was one of the key studies evaluating the efficacy and safety of canakinumab in a broad spectrum of autoinflammatory diseases. The study included 63 patients with FMF (29 children), 72 patients with MKD (54 children), and 46 patients with TRAPS (27 children). At week 16 (Epoch 2 phase), the proportion of patients exposed to canakinumab 150mg every 4 weeks with a complete response was significantly higher than in the placebo group: 61 vs. 6% for FMF (p <0.001), 35 vs. 6% for MKD (p = 0.003), and 45 vs. 8% for TRAPS (p = 0.006). The response rate further increased after dose escalation (canakinumab 300mg every 4 weeks) in patients who did not fully respond and led to complete response in 71% FMF patients, 53% MKD and 73% TRAPS patients. All responders in FMF group, 82% in MKD and 83% in TRAPS were still maintaining the responsiveness to canakinumab in prolonged intervals to 8 weeks (Epoch 3 phase). The infections were reported as the most common AEs, twelve of them were assessed as serious, however all of them resolved without sequelae. No opportunistic infections, cases of tuberculosis, or deaths occurred (55). Based on these significant and convincing results canakinumab has been approved for the FMF, MKD and TRAPS treatment.

### Registry-Based Studies

Van der Hilst et al. (56) et al. analyzed the response to various therapeutic modalities in a cohort of 103 HIDS patients from International HIDS registry. No or very limited response was observed in a patients treated with conventional therapeutics, such as MTX, AZA, SAS, tacrolimus, dapsone, IVIG, cimetidine, ranitidine as well as colchicine. Higher response rate was seen in 62.2% and 80% patients treated with high doses of GC and biologics—anakinra and etanercept. Patients with limited efficacy of anakinra responded to etanercept and vice versa (56).

Ter Haar et al. (93) aimed to evaluate the treatment response to IL-1 inhibitors in 496 patients from the EuroFever Registry (EFR). In this study, data on 121 FMF patients were analyzed and correlated with the evidence from the literature along with 94 CAPS patients, 113 TRAPS patients and 67 MKD patients from the EFR. Out of all registry, only three FMF patients from a cohort of 121 patients were treated with anakinra, in whom complete response (defined as absence of signs of active disease and reported normal levels of inflammatory markers) was achieved. In CAPS patients, the level of complete responses to anti-IL-1 therapy in the registry was far lower than the number of complete responses reported in the literature for anakinra (65% in EFR vs. 85% in the literature). In TRAPS patients, the response to anti-IL-1 treatment was comparable among both groups (registry patients vs. literature reports). Moreover, TRAPS patients tended to respond better to IL-1 targeted therapies than to anti-TNF agents. In contrast to the literature, a comparable response rate was further observed among MKD patients treated either with corticosteroids or Non-steroidal anti-inflammatory drugs (NSAIDs), anti-TNF, or anti-IL-1 agents. To conclude, the authors suggest IL-1 inhibitors are treatment of choice particularly in CAPS patients. On-demand use of GC and NSAIDs represents a possible therapeutic approach in patients with MKD and TRAPS as well as colchicine in FMF patients. In these patients IL-1 blockade should be indicated in poorly controlled patients with conventional therapy (93).

## Conclusion

Dysregulation of the IL-1 cytokine family leads to the development of multiple disorders, such as AIDs, which include periodic fever syndromes—CAPS, FMF, TRAPS, and MKD/HIDS. The significant therapeutic effect of IL-1 inhibitors in the treatment of AIDs highlights the crucial role of IL-1 cytokines in the disease pathogenesis. Recently, IL-1 inhibitors have become a breakthrough therapy in the treatment of AIDs.

We have, thus, reviewed the best available evidence on the IL-1 blockade in monogenic AIDs. Randomized clinical trials, as well as the registry-based trials and open-label studies were closely reviewed to bring more light into the potential of IL-1 inhibitors in the treatment of adult and pediatric AID patients.

Overall, IL-1 inhibitor monotherapy leads to a decrease in the disease activity and contributes to the reduction in or withdrawal of NSAID/corticosteroid therapy. The majority of the above discussed studies have proven that early IL-1 blockade may dramatically change the course of the disease, improve the quality of life of AIDs patients and prevent the attacks of disease. This is was found to be particularly relevant in FMF patients that are unresponsive to first line therapies, such as colchicine therapy. However, colchicine is advised to be continued in patients recieving biologic drugs.

IL-1 inhibitors were found to display only limited efficacy in the treatment of secondary AID-induced amyloidosis and articular involvement. Moreover, to prevent disease flares and severe complications, long-term administration of IL-1 inhibitors is usually required.

TNFα inhibitors, on the other hand, treat efficiently articular manifestations but often fail to reduce systemic features in monogenic AIDs.

Apart from clinical response, a reduction of laboratory inflammatory markers was notable in most of the studies. Among the inflammatory parameters, CRP, ESR and SAA were routinely examined in most of the patients, and together with other inflammatory parameters, such as S100 proteins (S100A12, calprotectin) and D-dimers, presented a valuable tool for the disease monitoring.

IL-1 inhibitors have been shown to have good safety profiles in open-label and randomized clinical trials. Furthermore, the favorable safety profiles of canakinumab and anakinra were also confirmed in the registry-based and retrospective studies reflecting the “real life” evidence.

The most common adverse events were infections and local skin reactions at the injection site, particularly in patients treated with anakinra. These adverse events, however, especially in pediatric patients, often appeared as a reason for therapy discontinuation. In point of fact, in most of the studies with pediatric patients, the main reason for the therapy switch to canakinumab was the daily administration on anakinra. Therefore, it was the burden of anakinra daily administration rather than the loss of efficacy that favored canakinumab over anakinra in selected studies.

Although both anakinra and canakinumab are currently the most effective treatments for AIDs, to date, there are no prospective or controlled head-to-head trials that would conclude the superiority of one anti-IL-1 therapy over another.

The high cost of canakinumab therapy remains a concern and could favor anakinra in the treatment of adult AIDs patients. On the contrary, the current evidence suggests that canakinumab is better tolerated in pediatric patients due to the lower frequency of therapy administrations. Rilonacept also represents a treatment of choice in selected diseases; however, it is currently only FDA approved in the United States for the treatment of CAPS.

Several clinical trials are currently ongoing to test IL-1 inhibitors and drugs with different mechanisms of action, such as tadekinig alfa, dapansutrile and tranilast. Moreover, novel compounds, such as peptides and small-molecule inhibitors of the NLRP3 inflammasome, have been shown to serve as modulators of the IL-1 mediated inflammation and are currently tested in multiple pre-clinical studies.

Despite so far satisfactory results with anakinra and canakinumab in the treatment of AIDs, the therapeutic potential of other IL-1 targeting therapies is yet to be revealed.

## Author Contributions

HM: The main author, she contributed in literature review and summary, manuscript drafting (the sections Methods, Cryopyrin-Associated Periodic Syndrome, TNF Receptor-Associated Periodic Syndrome, Familial Mediterranean Fever, Mevalonate Kinase Deficiency/Hyper IgD Syndrome, and Conclusion). ZS: The main author, she contributed in literature review and summary, manuscript drafting (the sections Introduction, Cytokines of the IL-1 Family, and Inhibitors of the IL-1 Cytokine Family). TM: The corresponding author, he contributed in data curation and validation, manuscript review, and editing. IS: The (senior) co-author, he contributed in manuscript critical review (the sections Introduction, Cytokines of the IL-1 Family, and Inhibitors of the IL-1 Cytokine Family). AS: The (senior) co-author, he contributed in manuscript critical review (the sections Methods, Cryopyrin-Associated Periodic Syndrome, TNF Receptor-Associated Periodic Syndrome, Familial Mediterranean Fever, Mevalonate Kinase Deficiency/Hyper IgD Syndrome, and Conclusion). RH: The (senior) co-authors, he contributed in manuscript conceptualization, critical review, and supervision. All authors contributed to the article and approved the submitted version.

## Funding

The preparation of this manuscript was supported by the grant of the Czech Health Research Council, the Ministery of Health, the Czech Republic nr. NU20-05-00320

## Conflict of Interest

The authors declare that the research was conducted in the absence of any commercial or financial relationships that could be construed as a potential conflict of interest.
